# Salivary Cortisol Reactivity in Preterm Infants in Neonatal Intensive Care: An Integrative Review

**DOI:** 10.3390/ijerph13030337

**Published:** 2016-03-18

**Authors:** Evalotte Mörelius, Hong-Gu He, Shefaly Shorey

**Affiliations:** 1 Department of Social and Welfare Studies, Division of Nursing Science, Linköping University, Norrköping 60174, Sweden; 2 Alice Lee Centre for Nursing Studies, Yong Loo Lin School of Medicine, National University Health System, National University of Singapore, Singapore 119077, Singapore; nurhhg@nus.edu.sg (H-G.H.); nurssh@nus.edu.sg (S.S.)

**Keywords:** cortisol, infants, neonatal care, nursing, pain, preterm, saliva, stress

## Abstract

Recently, more and more researchers have been using salivary cortisol reactivity to evaluate stress in preterm infants in the neonatal intensive care unit (NICU). The aim of this integrative literature review was to summarize the evidence of interventions leading to a change in salivary cortisol from the baseline in preterm infants in the NICU. The electronic databases of PubMed, CINAHL, Web of Science, and Scopus were searched for relevant studies. The inclusion criteria were studies with preterm infants exposed to an intervention evaluated by salivary cortisol reactivity before discharge from the NICU, which were published in English. In total, 16 studies were included. Eye-screening examination and heel lance provoked an increase in the salivary cortisol level. Music, prone position, and co-bedding among twins decreased the salivary cortisol level. Several studies reported a low rate of successful saliva sampling or did not use control groups. Future studies need to focus on non-painful interventions in order to learn more about salivary cortisol regulation in preterm infants. Moreover, these studies should use study designs comprising homogenous gestational and postnatal age groups, control groups, and reliable analysis methods that are able to detect cortisol in small amounts of saliva.

## 1. Introduction

The hypothalamic–pituitary–adrenal (HPA) axis regulates cortisol production and the organism's capacity to respond to stressors and thus maintain homeostasis. From the beginning of the second trimester, the HPA axis functions and organizes in response to the environment [1,2,3]. Secretion of the cortisol is essential for lung maturation at birth, hence, there is a corresponding increase in cortisol levels with increasing gestational age [4,5,6]. One month after birth, full-term healthy infants develop a circadian rhythm of salivary cortisol with increasing morning levels and corresponding decreasing evening levels [7]. In preterm infants, developmental immaturity and/or the effects of critical illness on adrenal function may result in insufficient cortisol production to maintain homeostasis when exposed to a stressor [8,9]. Thus, preterm infants may be able to produce enough cortisol to maintain homeostasis under non-stressful conditions, but insufficient cortisol to respond appropriately when exposed to a stressor. An appropriate cortisol release in the face of a stressor is vital for survival and the lack of such a response increases the risk of morbidity and mortality in preterm infants [1]. On the other hand, longstanding high levels of cortisol may increase the risk of cognitive and behavioral problems, high blood pressure, and development of the metabolic syndrome [10,11,12,13,14].

The life of the preterm infant is inherently stressful from the moment of birth as the environment in a NICU is usually busy. Several infants are cared for in the same room; sounds and alarms from cardio respiratory monitors, incubators, and mechanical ventilators add to their stress. As a part of their medical care, the preterm infants are not only subjected to many different invasive procedures but also bombarded with stimuli from daily handling procedures (for instance: diaper changing, repositioning, weighing, and personal hygiene care). In a Canadian observational study, Johnston and colleagues found that preterm infants were subjected to a mean of six tissue-damaging and 14 non-tissue-damaging procedures in one week [15]. Grunau and colleagues have shown that children born very preterm exhibit altered HPA axis functioning at three, eight, and 18 months, and at seven years [16,17]. Moreover, they found that elevated salivary cortisol levels in eight and 18-month old preterm infants positively correlated to the amount of pain exposure in the neonatal period [18,19,20]. Compared to full-term infants, preterm infants are at greater risk of short-term consequences of stress (e.g., fluctuations in intracranial blood pressure with an increasing risk for intraventricular hemorrhage [21,22]), as well as long-term consequences of stress (e.g., allostatic load and an inability to respond appropriately to a stressor [23]).

In a review from 2009, it was found that infants aged under three months showed increased cortisol levels from baseline in response to painful interventions (e.g., heel lance and inoculation) as well as routine handling interventions (e.g., physical examination, diaper changing and removal from bath) [24]. Several studies indicated that in relation to routine handling interventions, preterm infants had symptoms of stress such as increased heart rate, skin conductance, and pain scores, and decreased oxygen saturation [25,26,27,28,29]. Pleasant interventions such as getting a massage and listening to recorded sounds of the maternal heartbeat have been shown to decrease salivary cortisol levels in full-term infants [30,31]. A combination of sweet-tasting oral solution and pacifier as pain relief during vaccination has also been shown to decrease salivary cortisol levels in full-term infants [32], as has non-nutritive sucking during circumcision [33]. Moreover, practicing neonatal care methods such as family-centered care, skin-to-skin contact (SSC) and holding have been proved to have a beneficial impact on the developing HPA axis in preterm infants [34,35,36]. However, no review has summarized preterm infants’ cortisol reactivity in response to painful, handling and pleasant interventions, respectively.

Mode of delivery and prenatal exposure to maternal stress have also been shown to affect the subsequent cortisol reactivity of the infant [37,38,39]. For example, Taylor and colleagues found a lower cortisol reactivity during a four-month-vaccination among infants delivered by cesarean-section compared to vaginally-born infants [37]. Similarly, O'Connor and collaborators found lower cortisol reactivity during stress provocation (Ainsworth’s strange situation) in 17-month-old infants who were exposed to high levels of cortisol *in utero* [39].

It is important to know what stressors are suitable to use to evaluate salivary cortisol reactivity in preterm infants in order to guide researchers in future studies. Available reviews were focused on salivary cortisol reactivity in response to acute stressors in adults [40] and children [24,41]. Cortisol in saliva has been used as a valid method to measure adrenocortical activity in newborns since 1987 [42]; however, no reviews have been conducted to summarize the salivary cortisol reactivity in relation to acute stressors in preterm infants in the NICU. Moreover, it is important to evaluate the type of interventions performed in the NICU that are beneficial for preterm infants in order to prevent longstanding high levels of cortisol and allostatic load.

The aim of this review was to summarize the evidence from interventions leading to a change in salivary cortisol levels from the baseline (before the intervention) to response (after the intervention) in preterm infants in the NICU. The specific research questions were:

- What interventions lead to an increase in salivary cortisol levels from the baseline to response in preterm infants in the NICU?
- What interventions lead to a decrease in salivary cortisol levels from the baseline to response in preterm infants in the NICU?

## 2. Materials and Methods 

### 2.1. Design

This was an integrative literature review, which provided a summary of the interventions leading to either an increase or decrease in salivary cortisol levels from the baseline in preterm infants in the NICU. 

### 2.2. Study Selection

The inclusion criteria were studies with preterm infants exposed to an intervention evaluated by salivary cortisol reactivity (baseline and response values) in the NICU. Studies without an intervention or studies using adrenocorticotropic hormone (ACTH) stimulation, studies with only baseline or only response values presented, and successful saliva sampling ≤ 50% were excluded. Studies using citric acid as a saliva stimulant were also excluded since this is known to interfere with the cortisol levels by lowering the sample PH [43]. 

### 2.3. Search Strategy

Four electronic databases (PubMed, CINAHL, Web of Science, and Scopus) were used to search for relevant studies. The search terms used were *cortisol, infant, saliva/salivary, and preterm/premature*. Limits were set to English and human, and no limit was set for years. The search took place in September 2015. 

**Figure 1:**
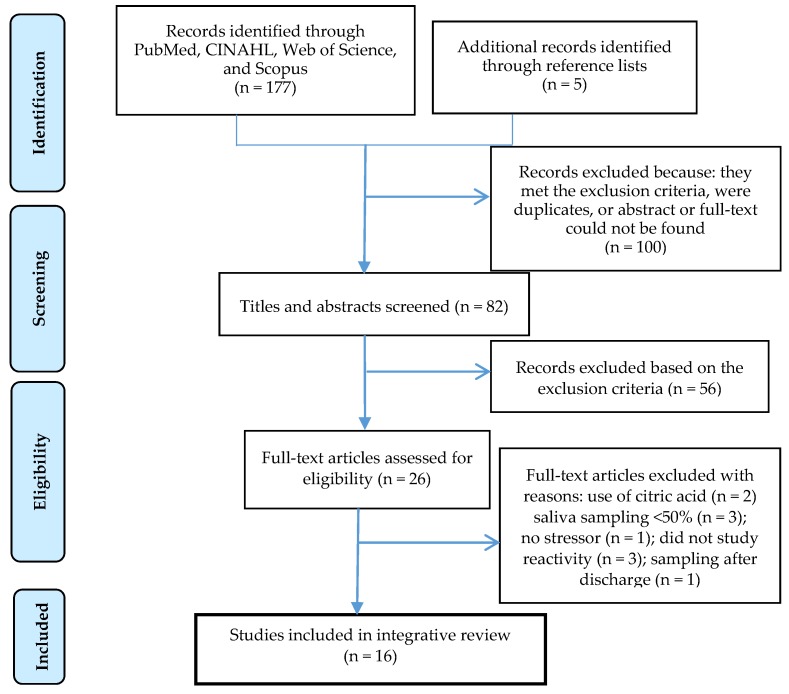
PRISMA flow diagram on the outcomes of the search strategies.

The database search resulted in 177 articles. Additional studies were also identified through other sources e.g., reference lists (*n* = 5). Abstracts were reviewed (*n* = 82) and all articles that could not be conclusively excluded based on the abstract were retrieved to determine eligibility. After full-text reading (*n* = 26), 10 articles were excluded due to the exclusion criteria (Figure 1). Eventually, 16 studies were included in this review (Table 1). 

### 2.4. Analysis Method

The effect size was not calculated in this integrative review since less than half of the studies presented mean and standard deviations while the other studies presented median values or just showed the cortisol levels in graphs. Instead, we examined and summarized whether the change in salivary cortisol from the baseline to response was statistically significant or not, and if so, in which direction [24]. The directions were classified as an increase or decrease. If the study involved the presentation of salivary cortisol results for both preterm and full-term infants, information from the preterm infants was extracted. 

## 3. Results

### 3.1. Characteristics and Quality of Included Studies

Table 1 shows the descriptive data of the included studies. Ten studies included infants born ≤28 weeks gestational age (GA) [29,47,48,49,50,51,54,55,56,58]. Of the studies comparing data between two groups, four studies recruited ≥50 infants in each group [46,47,51,54]. All studies but one [57] collected response samples at the recommended time of 20–30 min [59]. Several studies reported problems with saliva collection from preterm infants; six studies reported a successful saliva sampling rate ≥90% [29,44,50,53,54,58]. Five studies [44,46,47,52,55] did not state the storage temperature of the saliva samples and one study [51] reported a storage temperature above the recommended −20 °C, which may have increased the risk of evaporation [60]. Two studies did not report the analysis methods for salivary cortisol [45,51]. Seven studies did not report the coefficient of variation for the methods used [44,46,47,51,55,57,58], which makes it more difficult to interpret the quality of the analysis method.

Among the seven studies comparing salivary cortisol reactivity between two different preterm groups, three studies included the mode of delivery as a background variable [48,53,54]. One study found a significant difference in the mode of delivery between groups but did not control for mode of delivery in the statistical analysis of cortisol reactivity [48]. All studies except two [52,53] reported obtaining approval from an ethical board.

### 3.2. Interventions Leading to a Change in Salivary Cortisol from Baseline 

The interventions that were found to lead to an increase in salivary cortisol levels from the baseline were the heel lance, physical examination, and eye-screening examination (Table 2). The interventions that were found to lead to a decrease in salivary cortisol levels from the baseline were prone position and live harp music (Table 3). However, the heel lance and eye-screening examination also led to a decrease in cortisol when non-pharmacological methods aiming to reduce pain were used, *i.e.*, co-bedding of twins or behavioral support according to the Newborn Individualized Developmental Care and Assessment Program (NIDCAP) (Table 3). The interventions used in the reviewed studies are classified as painful, handling, or pleasant in this report. 

### 3.3. Summary of Painful, Handling, and Pleasant Interventions

#### 3.3.1. Painful Interventions

The painful interventions from the reviewed studies included the heel lance, eye-screening examination, nasopharyngeal suctioning, and “different painful procedures”. Seven studies used the heel lance as a painful intervention [44,45,46,47,48,52,53]. Two of these studies reported significant increases from the baseline in salivary cortisol levels in preterm infants born >30 weeks GA [52,53]. Magnano and colleagues compared 11 infants exposed to cocaine *in utero* with 47 infants not exposed to cocaine and found an increase in salivary cortisol level in response to the heel lance in both groups [52]. Davis *et al.* compared nine infants exposed to antenatal betamethasone with nine unexposed infants and found a significant increase in salivary cortisol levels in response to the heel lance among the unexposed infants and a significant decrease in salivary cortisol levels among the betamethasone-exposed infants [53] (Table 2). A significant decrease in salivary cortisol levels in relation to the heel lance was reported by Campbell-Yeo *et al.* [48]. They compared twins exposed to the heel lance whereby one group was randomized to co-bedding at least 24 h before the heel lance. Regardless of group assignment, all infants received one dose of oral 24% sucrose 2 min before the heel lance and were offered a pacifier. The results showed a significant decrease in salivary cortisol levels in the twins having the co-bedding experience (*n* = 72) but not in the control group (*n* = 62) (Table 3). One article using the heel lance as an intervention found no significant changes in salivary cortisol levels when administrating pain relief, *i.e.*, oral sucrose, to the infants [44] (Table 1).

Three studies reported differences between the intervention and control groups in salivary cortisol levels after the heel lance but did not provide significance levels for cortisol reactivity for each group [45,46,47]. Cong *et al.* performed a crossover trial comparing infants when they were exposed to SSC prior to and during the heel lance with incubator care [45]. They found that 20 min after the heel lance, infants exposed to SSC for 30 min (*n* = 18) had lower salivary cortisol compared to those in the control group whose heel lance was performed in the incubator [45]. Badiee *et al.* compared infants either exposed to formula milk odor or breast milk odor during the heel lance. Infants exposed to maternal breast milk odor (*n* = 25) had significantly lower salivary cortisol levels 20 min after the heel lance compared to infants exposed to formula milk odor (*n* = 25) [46]. In 2014, Badiee and her colleagues performed another trial with twins randomized to co-bedding in the incubator or separate bedding. Infants co-bedded 24 h prior to the heel lance (*n* = 50) had significantly lower salivary cortisol levels 20 min after a heel lance compared to infants randomized to separate bedding (*n* = 50) [47] (Table 1). 

Salivary cortisol reactivity has also been studied in relation to “different painful procedures” (e.g., heel lance, venipuncture, and suctioning) [51], nasopharyngeal suctioning [50], and the eye-screening examination [49] (Table 1). The first two studies reported no significant change in cortisol levels between the baseline and response. Kleberg *et al.* performed a randomized crossover trial with 36 infants randomly assigned at the first eye-screening examination to receive either behavioral support according to NIDCAP or standard care. The eye-screening examination provoked an increase in salivary cortisol from the baseline at both 30 and 60 min after the examination in both groups [49] (Table 2). 

#### 3.3.2. Handling Interventions

The handling interventions from the reviewed studies included physical examination, prone position, and diaper change. Five studies used handling as an intervention [29,52,53,54,55]. Magnano and colleagues found a significant increase in salivary cortisol in non-cocaine-exposed infants (*n* = 47) in response to physical examination but not for cocaine-exposed infants (*n* = 11) [52] (Table 2). Davis *et al.* reported non-significant results in response to physical examination in both antenatal betamethasone-exposed infants (*n* = 9) and non-exposed infants (*n* = 9) [53] (Table 1). 

Changing to the prone position was used as an intervention in one study [55]. In a single group (*n* = 21), Candida *et al.* found a significant decrease in salivary cortisol 30 min after changing infants’ position from the lateral/supine position to the prone position in the incubator [55] (Table 3).

Mörelius and colleagues compared preterm infants (*n* = 30) with full-term infants (*n* = 39) in response to a standardized diaper change performed by staff during the first and the fourth weeks of life. There were significant differences in cortisol levels between preterm and full-term infants but no significant difference between baseline and response values [29]. In 2012, Mörelius and her colleagues again used a diaper change as an intervention, but this time the mother changed the diaper instead of staff. Infants randomized to a family-centered NICU (*n* = 152) where the parents stayed around the clock were compared with infants in a ward where the parents had to sleep at home (*n* = 132). No significant differences in salivary cortisol reactivity were found in either group [54].

#### 3.3.3. Pleasant Interventions

The pleasant interventions from the reviewed studies included music, acoustic stimulation, maternal voice, and SSC. Three studies used pleasant interventions [56,57,58]. Schwilling *et al.* [58] introduced a single group of infants (*n* = 20) to live harp music for 15 min a day for three consecutive days and found decreased salivary cortisol levels in response to the music on days one and three but not on day two. The salivary cortisol level was also significantly lower than the baseline 4 h after exposure to music during day one but not on days two and three (Table 3). Similarly, Dorn and colleagues [57] studied the influence of acoustic stimulation and randomized 60 infants to three groups; audiotaped lullabies, maternal voice, and control. Infants in the audiotaped lullabies and maternal voice groups were exposed to acoustic stimulation for 30 min every day over a period of two weeks. No significant changes in salivary cortisol reactivity were found. One study investigated salivary cortisol reactivity during SSC. Cortisol was measured at three time points (in the incubator, during SSC, and back in the incubator) on two different occasions (first and fourth SSC). No significant results in cortisol reactivity were found [56] (Table 1). 

## 4. Discussion

This review summarizes the evidence of painful, handling, or pleasant interventions that caused a change in salivary cortisol from baseline in preterm infants in the NICU. 

Two studies reported significantly increased salivary cortisol levels among preterm infants after exposure to a painful procedure [49,52]. These two studies, which recruited preterm infants born at 23–37 weeks GA, reported a successful saliva sampling above 75% and showed significant results despite relatively small sample sizes [49,52]. The result is congruent with previous studies, which found that healthy infants usually show an elevation in cortisol in response to painful procedures [24,41]. A reaction to pain is a defense mechanism aiming to protect the person from harm and to maintain homeostasis, and strong stimuli such as pain tends to yield non-habituation in healthy infants [61,62].

It is well known that pain relief such as sweet-tasting oral solutions and non-nutritive sucking have proven to be effective in reducing pain during single painful procedures in newborns [63,64,65,66]. The administration of optimal pain relief probably explains the absence of salivary cortisol reactivity in response to the heel lance in the study by Cignacco *et al.* [44]. In the study of Campbell-Yeo *et al.* [48], infants were given oral sucrose and a pacifier before the heel lance, and co-bedded twins experienced a decrease in salivary cortisol levels, but not separated twins. A dampened cortisol response in infants receiving a pacifier and a sweet-tasting oral solution during a painful procedure is likely to reflect an absence or a lower degree of pain and stress. A significant decrease in response to the heel lance without any pain relief as with the infants exposed to antenatal betamethasone in the study by Davis *et al.* [53] is more likely to be an effect of suppressed adrenal activity due to the corticosteroid use [67], which is not the same as no pain or stress. It is also possible that co-bedded twins were experiencing relief due to the reunion, which affected the adrenocortical response through human touch and closeness [34,36,68]. Thus, it is possible that the cortisol level decrease was exclusively due to co-bedding the twins. However, it is noteworthy that the nurses who performed the heel lances in the study by Campbell-Yeo *et al.* were not blinded to group assignment and the successful saliva sampling was rather low [48]. Badiee *et al.* revealed similar results. They found a significantly higher cortisol level after the heel lance in the standard care group compared to the co-bedding group, but did not provide cortisol reactivity results [47]. However, the results are promising and, therefore, the replicability of these findings needs to be further investigated. Kleberg *et al.* used behavioral support but found no effect of NIDCAP on cortisol levels during the eye-screening examination. However, they found a more rapid recovery in the NIDCAP group [49]. Recovery, the degree to which cortisol elevations persist after termination of the stressor [69], was studied in only two of the included articles [49,53]. Since preterm infants are subjected to several procedures each day, it is important to continue to investigate the recovery from stressful stimuli in preterm infants in order to learn more about their stress system development and to be able to provide optimal support in the NICU. 

Few reviewed studies used handling as a stressor [29,52,53,54,55]. Magnano and colleagues found that healthy preterm infants did not respond to a physical examination while antenatal cocaine-exposed infants’ salivary cortisol levels increased [52]. This result is in accordance with the theory of habituation. Repeated milder stressors that do not involve a threat to physical or psychological well-being may yield habituation of the adrenocortical response in healthy infants but not necessarily in unhealthy infants [61,62]. 

It is of the utmost importance to find care methods that can buffer stress and, thus, longstanding elevated levels of cortisol in preterm infants in the NICU. Two studies found decreased salivary cortisol levels in response to a changed positioning from lateral/supine to prone and live harp music, respectively [57,58]. However, both studies used the single group method, which makes it difficult to determine whether the decrease in cortisol levels was due to the intervention or a natural decline in cortisol levels [7]. Moreover, coefficients of variation for the assay methods were not reported, which affects the reliability. Since the results are important and interesting, both studies should be repeated using a randomized, controlled study design. 

An absence of cortisol response in a stressful situation which may threaten the homeostasis of the premature infant may have several causes. First, suppressed adrenal activity due to corticosteroid treatment [67] or longstanding high stress load leading to allostatic load and an inability to respond [23]. Second, a high baseline value, resulting in a smaller rise as explained by the law of initial value [70]. Third, immaturity of the HPA axis system causing inconsistent, less robust cortisol responses [4]. Fourth, the stressor is too mild and/or the infant has become habituated to the procedure [62]. Fifth, methodological issues such as study design, lack of power, saliva collection time, and quality of the cortisol analysis method. 

In this review, several studies reported an absence of cortisol response in relation to painful procedures [44,50,51], handling [29,54], and pleasant interventions [56,57]. Boyer *et al.* presented some methodological issues with low successful saliva sampling and no report of analysis method [51]. Dorn *et al.* [57] reported a rather low rate of successful saliva sampling, no intra-assay coefficient of variation, and moreover collected response values after 10 min instead of the recommended 20–30 min [59]. In studies by Ivars *et al.* and Mörelius *et al.* 2006 and 2012, it is plausible that the stressors were too mild for rather stable infants, and so the studies ought to be repeated with more well-defined groups comprising only infants with low GA and low postnatal age [29,50,54]. If the parent is performing the diaper change, it is also important to control for parental sensitivity and SSC, since both parental sensitivity and SSC may buffer the infant’s stress reaction [34,36,71]. In 2005, Mörelius *et al.* included infants with a wide range of GA from 25 to 33 weeks during SSC [56]. The results show a large variability in the cortisol responses with both elevated and decreased levels reflecting an immature HPA axis. Several studies included in this review examined an unselected group of preterm infants in terms of GA. Extremely preterm infants are more immature and there is no correlation between ACTH and cortisol as there is in more mature infants [19]. Hence, researchers should consider the GA of preterm infants when designing studies of salivary cortisol reactivity in the future, and should aim at creating more homogenous groups for better comparison. 

Other methodological issues such as obtaining optimal sample volumes of saliva without using a saliva stimulant could be reasons for the small number of studies performed in the NICU. A sampling time exceeding 5 min [57], cheek massage [48], or aspiration of saliva with a syringe [53,55] may be stressors in themselves and may confound the cortisol results. It is therefore valuable to adopt non-invasive sampling methods as well as measurement methods using minute sample volumes when studying salivary cortisol in preterm infants [72,73,74]. 

In this review, we have summarized the cortisol reactivity in relation to different interventions but we have not evaluated the interventions per se. Thus, it is possible that the included interventions, e.g., diaper change and SSC, may still be stressful or have a calming effect for the preterm infants in the NICU even though there was no change in cortisol levels. Studies that did not report ethical approval were also included in this review and the advisability of such inclusion may be debatable. However, the reasons for inclusion of these studies were that both studies used stressors that were part of the infants’ care and the results are valuable for the understanding of stress reactivity in preterm infants. 

## 5. Conclusions 

Salivary cortisol has been used as a marker of stress in preterm infants in the NICU since 1992. This integrative review showed that painful interventions, such as the heel lance and eye-screening examination, could lead to an elevation in salivary cortisol levels from the baseline in preterm infants in the NICU. Pain relief and antenatal glucocorticoids may dampen the response for different reasons. Changing to the prone position and exposure to live harp music were promising interventions that could lead to a decrease in salivary cortisol levels from baseline. However, these interventions need to be further investigated with designs comprising control groups. Several studies report a low percentage of successful saliva sampling. For future studies, it is important to utilize analysis methods using minute saliva sample volumes in order to increase reliability. In addition, the stressfulness of many interventions routinely performed in the NICU has never been evaluated with salivary cortisol, and future studies should focus on interventions that involve the infant and the infant’s nest such as changing of cot-sheets, personal hygiene care, and separation from the parent.

## Figures and Tables

**Table 1:** Descriptive data of included studies (n = 16).

Author Year Country	GA Age ^+^	Post–Natal Age, Days	Design	Study Groups	*n*	**Intervention**	**Sampling Times**	**Successful Saliva Sampling**	**Storage Temperature (°C)**	**Analyze Method**	**Intra/Inter CV**	**Cortisol Response**
Painful												
Cignacco *et al.* 2009 Switzerland [44]	28–31	<14	Single group	Sucrose	9	Heel lance	–25, +30	99%	Not stated	RIA	Not stated	Not significant
Cong *et al.* 2011 USA [45]	30–32	2–9	Randomized crossover	Incubator care *vs.* SSC 30 min or 80 min	18 + 10	Heel lance	0, +20	*	−70	Not stated	4.8/6.3	Not stated
Badiee *et al.* 2013 Iran [46]	32–37	1–30	Randomized clinical study	Formula odor *vs.* breast milk odor	25 + 25	Heel lance	0, +20	Not stated	Not stated	ELISA	Not stated	Not stated
Badiee *et al.* 2014 Iran [47]	26–34	<20	Randomized clinical study	Standard care *vs.* Co-bedding	50 + 50	Heel lance	0, +20	Not stated	Not stated	ELISA	Not stated	Not stated ^##^
Campbell–Yeo *et al.* 2014 Canada [48]	28–36	Mean 12.0 and 18.96	Randomized clinical study	Standard care *vs.* Co-bedding	62 + 72	Heel lance	0, +20	57%	−20	EIA	2.24/2.47	Decrease ^##^
Kleberg *et al.* 2008 Sweden [49]	23–31	<35	Randomized crossover	Standard care *vs* NIDCAP	36	Eye-screening exam.	0, +30, +60, +4 h	75%	−22	RIA	/6.0 –12.0	Increase ^#^
Ivars *et al.* 2012 Sweden [50]	27–33	4–86	Randomized crossover	Control *vs* oral glucose	11	Nasopharyngeal suctioning	0, +30	98%	−22	RIA	/6.0 –12.0	Not significant
Boyer *et al.* 2004 Canada [51]	<31	1–7	Randomized controlled study	Water *vs.* Sucrose for one week	105	Different painful procedures	0, +30	54%	−10	Not stated	Not stated	Not significant
Painful + Handling												
Magnano *et al.* 1992 USA [52]	30–37	5–53	Two group comparative design	Control *vs.* Cocaine exposed infants	47 + 11	Heel lance and Physical exam.	0, +30	79%	Not stated	RIA	3.3/10.1	Increase ^#^ and Increase ^#^
Davis *et al.* 2004 USA [53]	33–34	3–6	Two group comparative design	Control *vs.* Antenatal Betam-ethasone	9 + 9	Heel lance and Physical exam.	0, +20–25, +40–45	98%	−20	DELFIA	4.3/11.99	Decrease ^##^ and Increase ^#^
Handling												
Mörelius *et al.* 2006 Sweden [29]	23–38	2–7 and 10–18	Two group comparative design	Full-term *vs* preterm	39 + 30	Diaper change	0, +30	90%	−22	RIA	/6.0–12.0	Not significant
Mörelius *et al.* 2012 Sweden [54]	24–37	≤112	Randomized clinical study	Standard care *vs.* family care	137 + 152	Diaper change	0, +30	97%	−22	RIA	/6.0–12.0	Not significant
Candia *et al.* 2014 Brazil [55]	26–36	1–33	Single group	Lateral/supine position *vs.* Prone position	21	Prone position	0, +30	76%	Not stated	ECL	Not stated	Decrease ^##^
Pleasant												
Mörelius *et al.* 2005 Sweden [56]	25–33	2–21 and 4–26	Crossover	Incubator care *vs.* SSC	17	SSC	0, +30	89%	−22	RIA	/6.0–12.0	Not significant
Dorn *et al.* 2014 Germany [57]	30–37	Mean 4.9	Randomized clinical study	Control *vs.* Music *vs.* Maternal voice	22 + 20 + 20	Music and Maternal voice	−10, +10	65%	−20	ELISA	Not stated	Not significant
Schwilling *et al.* 2015 Germany [58]	23–33	<21	Single group	Day 1 Day 2 Day 3	20	Music	0, +25, +4 h	91%	−20	Mass- spec-trometry	Not stated	Decrease ^##^

^+^ Gestational age in weeks at birth; ^#^ See Table 2 for details; ^##^ See Table 3 for details; * Only reports how many subjects provided complete set of data, not how many were included. RIA = Radioimmunoassay; ELISA= Enzyme-linked immunosorbent assay; ECL = Electrochemiluminescence; EIA = Sensitive enzyme immunoassay; DELFIA = Competitive solid phase time-resolved fluorescence immunoassay with fluorometric endpoint.

**Table 2:** Studies reporting an increase in salivary cortisol levels from the baseline with different interventions (*n* = 3).

Author Year	Intervention	Results
Magnano 1992 [52]	Heel lance	Cocaine-exposed as well as non-cocaine-exposed infants showed a significant increase in salivary cortisol 30 min after heel lance.
	Physical examination	Non-cocaine-exposed infants showed a significant increase in salivary cortisol 30 min after physical examination.
Davis 2004 [53]	Heel lance	Infants not exposed to antenatal betamethasone showed a significant increase in salivary cortisol 20 min after heel lance.
Kleberg 2008 [49]	Eye-screening	NIDCAP treated as well as non-NIDCAP treated infants showed a significant increase in salivary cortisol from the baseline to 30 and 60 min after eye-screening examination for retinopathy of the premature eye.

NIDCAP = Individualized Developmental Care and Assessment Program.

**Table 3:** Studies reporting a decrease in salivary cortisol levels from the baseline with different interventions (*n* = 5).

Author Year	Intervention	Results
Davis 2004 [53]	Heel lance	Infants exposed to antenatal betamethasone showed a significant decrease in salivary cortisol from the baseline to 20 and 40 min after heel lance.
Kleberg 2008 [49]	Eye-screening examination	NIDCAP-treated infants showed a significant decrease in salivary cortisol from 30 to 60 min after eye-screening for retinopathy of the premature eye.
Campbell-Yeo 2014 [48]	Heel lance	Salivary cortisol was significantly lower than the baseline 20 min after heel lance in infants randomized to co-bedding but not in the control group.
Candia 2014 [55]	Prone position	Salivary cortisol was significantly lower than the baseline 30 min after changing the position from the lateral/supine position in the incubator to the prone position in the incubator.
Schwilling 2015 [58]	Music	Salivary cortisol was significantly lower than the baseline 25 min after exposure to 15 min of live harp music during days 1 and 3 but not on day 2. Salivary cortisol was significantly lower than the baseline 4 h after exposure to 15 min of live music during day 1 but not on days 2 and 3.

NIDCAP = Individualized Developmental Care and Assessment Program.

## Abbreviations

The following abbreviations are used in this manuscript:

ACTH	Adrenocorticotropic hormone
CV	Coefficient of variation
GA	Gestational age
HPA	Hypothalamic-pituitary-adrenal
NICU	Neonatal intensive care unit
NIDCAP	Newborn individualized developmental care and assessment program
SSC	Skin-to-skin contact
